# Biomass and mortmass of woody vegetation in metal-contaminated areas (Southern Urals, Russia)

**DOI:** 10.3897/BDJ.9.e75510

**Published:** 2021-11-29

**Authors:** Igor Bergman, Alexey Nesterkov

**Affiliations:** 1 Institute of plant and animal ecology, UB RAS, Ekaterinburg, Russia Institute of plant and animal ecology, UB RAS Ekaterinburg Russia

**Keywords:** aerial pollution, biodiversity, biomass, biotope, coarse woody debris (CWD), copper smelter, standing dead tree, downed bole, diameter at breast height (DBH), forest stand, mortmass, phytomass, stump, subcanopy

## Abstract

**Background:**

Since the mid-2000s, long-term monitoring of various components of natural ecosystems under conditions of industrial pollution has been carried out in the Southern Urals. As a part of these monitoring programmes, the data on various components of biota in different biotopes, collected with different methods and in different time intervals, continue to be gathered. In addition, data collected through these monitoring programmes can also be used to study the local biodiversity of non-polluted areas.

In 2012, in the vicinity of the Karabash Copper Smelter, a study of communities of small mammals was carried out, considering the heterogeneity of their habitats. Within the framework of this project, we presented a detailed description of the state of woody vegetation in the study area.

**New information:**

The dataset (available from the GBIF network at https://www.gbif.org/dataset/61384edd-2d0a-437b-8cf0-ff4d2dfcc0da) includes the results of an assessment of the woody vegetation biomass at seven habitats (pine, birch and floodplain forests, reed swamp, sparse birch stand, marshy meadow and dump of household waste) of areas with different levels of industrial pollution in the vicinities of the Karabash, the Southern Urals. Karabash Copper Smelter (KCS) is one of Russia’s most significant point polluters; the main components of its emissions are heavy metals, dust and sulphur dioxide. Parameters of woody vegetation (diameter at breast height, diameter at root collar level and biomass) were estimated for seven forest elements (forest stand, subcanopy (undergrowth and underwood), half-dead tree of a forest stand and four types of coarse woody debris (downed bole, fragment of downed bole, standing dead tree and stump)) at 41 sampling plots (20 at unpolluted and 21 at polluted areas) and 165 subplots (81 and 84, respectively). The dataset includes 411 sampling events (estimation events of the forest elements at sampling plots and subplots), corresponding to 5786 occurrences (estimations of the woody vegetation components) observed during July 2012. For most woody vegetation components (72%), an estimate of the above-ground phytomass is given. For each sampling event, information on the presence or absence of woody vegetation species at the considered habitats is provided (a total of 1479 occurrences with status "absent"). The dataset can be used for environmental monitoring, sustainable forest management, modelling forest productivity considering global changes, studying the structure and biodiversity of forest cover and assessing forests’ carbon-sequestration capacity. In addition, the dataset provides information about different forest ecosystems under the influence of strong industrial pollution.

## Introduction

To study the reaction of biota’s components to technogenic pollution, the biotope that dominates in the region and is represented in the entire pollution gradient is traditionally chosen. As a rule, this approach indicates an apparent decrease in biodiversity, abundance and biological productivity when proceeded towards the source of industrial emissions. However, in reality, the territories subjected to severe technogenic pollution are not a lifeless desert, but a mosaic of various biotopes (Mukhacheva et al. 2013). Therefore, studying the entire complex of biotopes of contaminated areas, one can come to fundamentally different conclusions of the biota’s response to pollution compared to the traditional approach. For example, in the near vicinities of the Karabash Copper Smelter (KCS), significantly degraded, almost lifeless areas coexist and alternate with relatively preserved biotopes that provide a wide range of small mammal species, although their numbers are reduced compared to non-polluted territories. It was shown that this environmental heterogeneity plays a crucial role in the small mammal’s diversity preservation under severe technogenic pollution (Mukhacheva et al. 2013); a similar result was obtained when studying the forest stands. An essential indicator of the forest stand’s state, the proportion of large trees (diameter at breast height is more than 20 cm) in pine and birch forests near the KCS was decreased as expected. However, on the contrary, in the floodplain stands of the polluted territory, the proportion of large trees increased and, in sparse birch stands, did not differ in the pollution gradient (Bergman et al. 2013).

The dataset includes information on the main woody vegetation parameters in seven types of habitats in areas with different levels of technogenic pollution. The presented data provide an opportunity to analyse the resistance of woody vegetation to technogenic pollution, considering the heterogeneity within the studied areas. The dataset contains the following parameters: species diversity and the number of woody plants, trunk diameters, above-ground phytomass (for living representatives) and mortmass of trunks (for dead representatives).

For most woody vegetation elements (72%) represented in the dataset, an estimate of the above-ground phytomass is given. The forest phytomass is the main parameter that determines the course of processes in forest ecosystems. It is used for environmental monitoring, sustainable forest management, modelling forest productivity taking into account global changes, studying the structure and biodiversity of forest cover and assessing forests’ carbon-sequestration capacity (Fowler et al. 2002).

The dataset provides information for studying the regularities of the biogenic carbon cycle as the main component of the global carbon cycle, the most crucial problem of modern ecology. This direction has acquired particular relevance in recent years in connection with regional and global climate changes. An assessment of the carbon balance is impossible without correct data on its content in each of the pools of the forest ecosystem, in particular, in the above-ground phytomass and mortmass of woody vegetation. For each of the biotopes presented in the dataset, coarse woody debris (CWD) is considered. CWD is understood as the dead matter of tree trunks (standing dead trees, downed boles, stumps) of all stages of decomposition, up to its transition to detritus. CWD is also a source of nutrients entering the soil, a source of food, a habitat for many species of animals, plants and fungi (Stokland et al. 2012, Bobkova et al. 2015). Interest in the study of CWD is not accidental and is due to its importance in the analysis of carbon sequestration processes (Karjalainen and Kuuluvainen 2002, Zamolodchikov 2009), as well as in the development of programmes for the conservation of forest ecosystem biodiversity (Siitonen 2001, Stokland et al. 2012). Considering, on the one hand, the apparent need for correct estimates of all components of the carbon balance and, on the other hand, the fact that industrially polluted areas are convenient for analysing the ecosystems resilience to strong impacts, the presented dataset has a “direct access” to operational issues of regional and global environmental changes discussed by the scientific community.

## Project description

### Study area description

The Ural Mountains are a north-south-orientated mountain system in the Urals, located between the East European and West Siberian plains. The study area is located in the lowest uplands (300–400 m altitude above sea level) in the southern taiga subzone. The prevailing forest types are herb pineries and secondary grass herb birch forests with linden, aspen and larch populations. The main soil types are brown mountain-forest and forest soils, grey soils, mountain-forest and mountain-podzol chernozems. The climate is continental, moderately cold. The annual temperature is 1.8°C and the average precipitation is 450–550 mm. The duration of the vegetation period is 160–170 days and the average height of the snow cover is about 40 cm.

**Figure 1.**
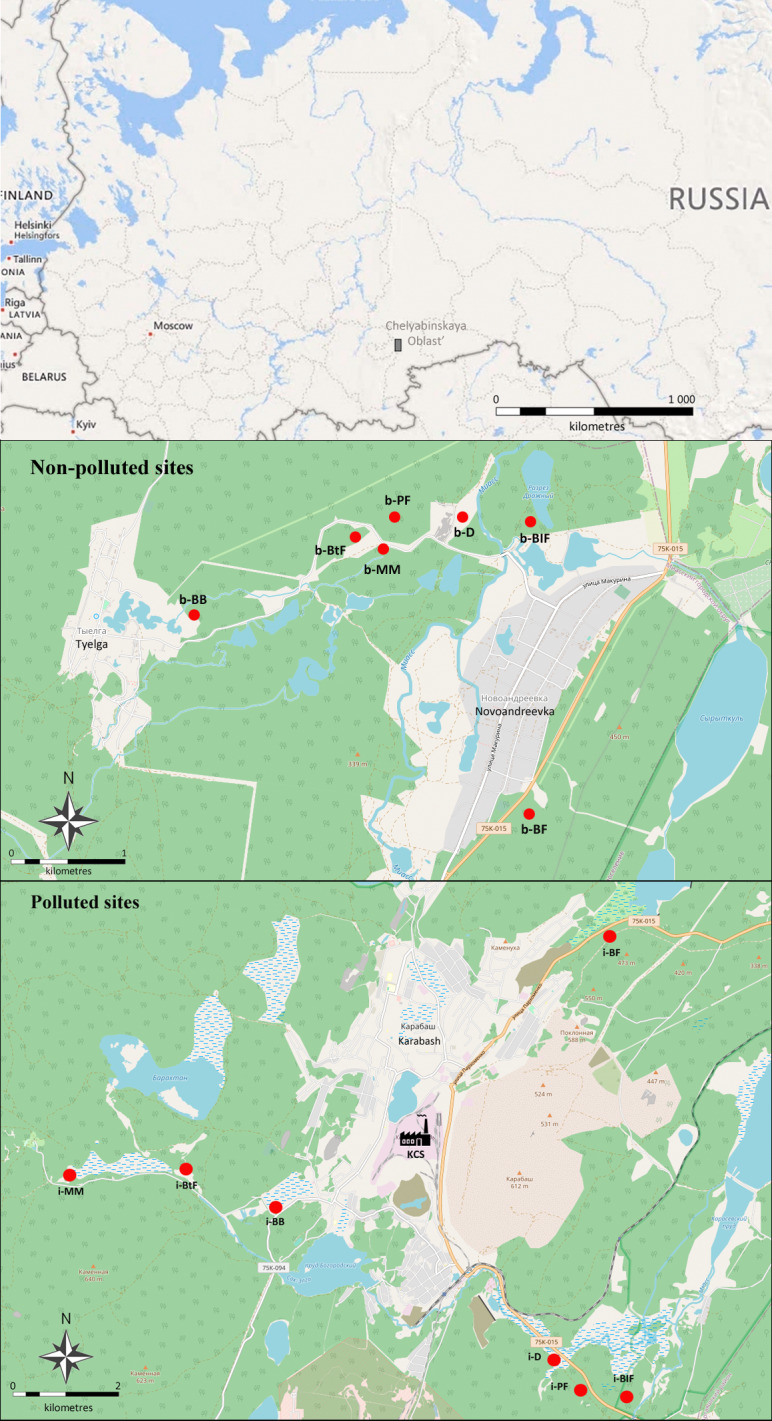
Location of the studied habitats at non-polluted and polluted areas in the vicinities of Karabash Copper Smelter, Southern Urals (data from Open Street Map; abbreviations of the habitats correspond to those in the dataset and Table 1).

The study was carried out in the Karabash Copper Smelter vicinities (Fig. 1), located within Karabash (90 km northwest of Chelyabinsk, Southern Urals) and it has operated since 1910. KCS is one of the largest point polluters in Russia; the main components of its emissions are heavy metals, dust and sulphur dioxide. The total amount of atmospheric emissions in the 1970s exceeded 370,000 t (of these, 364,500 t of sulphur dioxide and 28,800 t of dust, containing absorbed heavy metals, such as Cu (1530 t), Pb (2570 t) and As (1920 t)). In 2005, the total emissions were no more than 41,000 t, including 38,100 t of sulphur dioxide and 1300 t of dust containing 340 t of Cu, 20 t of Pb and 7 t of As. However, the pool of accumulated pollutants may still strongly impact the biota (Mukhacheva et al. 2010). Two study areas were explored: the first one with intense industrial pollution (0.5–5 km from the smelter, surveyed an area of about 30 km^2^; dead-cover forests with soil surface mostly covered with non-decomposed litter) and the second one with a regional background level of pollution (20–25 km south of the Smelter, total area of about 50 km^2^, forests with well-developed herbaceous layer (100% coverage in most cases)). Within each area, seven types of habitats were identified, differing in the position in the relief and vegetation structure.

## Sampling methods

### Study extent

The study was carried out in vicinities of the Karabash Copper Smelter (55.469 N, 60.209 E), located within the City of Karabash (90 km northwest of Chelyabinsk, Southern Urals). A total of 41 sampling plots were established in seven types of habitats: pine, birch and floodplain forests, reed swamp, sparse birch stand, marshy meadow and dump of household waste. All habitats were surveyed using six sampling plots, except for the reed swamp (five plots). The study was completed in July 2012.

### Sampling description

**The survey of forest woody vegetation at sampling plots**

The survey of forest woody vegetation is the leading and only method of accounting for forest resources, making possible the quantitative and qualitative evaluation of the forest health.

During the survey, we identified the following forest elements (according to Harmon et al. 2004):

1. the forest stand – living trees with a diameter at breast height (DBH) of more than 5 cm;
2. the subcanopy – all living trees with DBH of less than 5 cm and height of more than 10 cm; this forest element includes the undergrowth (trees, capable of forming the forest stand) and underwood (bushes, not capable of forming the forest stand);
3. the half-dead tree – the main trunk of which died, but branches with leaves emerge from the dormant buds preserved at the trunk. All detected trees were assigned to the forest stand;
4. the standing dead tree – dead but not fallen trees with a DBH no less than 5 cm and height more than 2 m;
5. the stump – lower part of the dead tree trunk, less than 2 m in height. The diameter was usually measured at the point of breaking of a tree trunk;
6. the downed bole – a fallen/hung tree trunk (or part of it), which is entirely within the sampling plot;
7. the fragment of a downed bole – a fallen tree trunk (or part of it), which is partially within the sampling plot (only the part within the plot was taken into account).

The information about the forest element type is encoded in the eventID and occurrenceID. The authors tried to establish the sampling plots so that the anthropogenic impact (recreation, grazing, felling, haymaking) was minimal. Three sampling plots with a size of 25 × 25 m were established within each type of habitat, except for the reed swamp (two plots at the unpolluted area, see Table 1). When the habitat configuration did not allow arranging a rectangular sampling plot, its size could vary within 471–691 m². Complete estimation of forest woody vegetation was performed at each site in both the subcanopy and forest stand. The trees in the forest stand were estimated throughout the entire sampling plot. An estimation of trees in the subcanopy was carried out at 1–10 subplots of 1–34 m^2^ selected within the sampling plot, depending on the biotope (Table 2).

The distance between the sampling plots in the birch, pine and floodplain forests and the sparse birch stand was 15–40 m. Therefore, subplots in these habitats were arranged randomly: the site was divided into equal squares 5×5 m (1×1 m for the sparse birch stand); each square was assigned a number. Then, three cards (except for some cases, see Table 2) with a number were selected randomly and the corresponding subplots within the sampling plot were approved. The reed swamp and marshy meadow were of limited size: in these habitats, the distance between sampling plots was several metres; in some cases, plots were adjacent to each other. The location of subplots within the sampling plot was uniform. For the waste dump, only one subplot within each sampling plot was established at places where above-ground vegetation was detected.

In the forest stand, a caliper with a scale up to 1 cm was used to measure the trunk diameters. Tree specimens with a diameter at breast height (1.3 m) equal to 5 cm or more were taken into account. Diameter measurements were taken in two mutually perpendicular directions, with the subsequent calculation of the arithmetic mean diameter. The diameters of the standing dead trees and half-dead trees were measured in the same way. The stump diameters were measured at the fracture point using a caliper in two mutually perpendicular directions. Stumps with a base diameter of more than 5 cm were taken into account. Next, the diameter of the subcanopy trees was measured at the level of the root collar using a pole caliper with an accuracy of 0.1 cm. Finally, the downed boles and fragments of downed boles were measured using a caliper: the base and the top diameters of the downed boles (cm) and its length (m) were measured.

The volume of downed bole (V_dbole_) was calculated using a modified truncated cone volume equation:

**V_dbole_=1/3π × H(R^2^_base_ + R_base_ × R_top_ + R^2^_top_)**,

where H – length of a downed bole, m; R_base_ – base radius of a downed bole, m; R_top_ – top radius of a downed bole, m (these three parameters are not included in the dataset, but are available upon request). The advantage of this formula is its simplicity (only three parameters are involved) and forest ecologists widely use it to assess downed bole biomass rapidly. The volume (m^3^) of each downed bole that falls within the sampling plot (a whole or a fragment) was calculated.

To calculate the above-ground phytomass of trees of the forest stand, subcanopy and standing dead trees in each habitat type, we used our data obtained and published earlier, which represent a detailed characterisation of the model trees (Usoltsev et al. 2012). A detailed description of the methods for determining the above-ground phytomass of model trees is given below. These data served as the basis for constructing regression equations and subsequent evaluation of the above-ground phytomass of each tree specimen in each habitat type.

For tree species of the forest stand, subcanopy and standing dead trees, the calculation of the parameters of the regression equations were performed by the formula (Kofman 1986):

**P_phytomass_=a_1_ × D**^**a**_**2**_^ ,

where P_phytomass_ – absolute dry above-ground phytomass of the plant, kg; D – diameter of the trunk, cm (for forest stand and standing dead trees measured at the height of 1.3 m, for subcanopy measured at root collar level); a_1_ and a_2_ – constants of the equation. This type of regression is considered to be the most biologically determined (Kofman 1986). Equation constants are selected by the non-linear estimation method (Levenberg-Macwardt algorithm). The procedure is implemented in STATISTICA v.8.0. Thus, the biomass of each woody plant can be efficiently and reliably estimated by the size of the trunk diameter using the presented dependence equations (Table 3).

Due to restrictions from the Forestry authorities, the following tree species were prohibited for felling: in the forest stand – *Acernegundo*, *Alnusincana*, *Crataegussanguinea*, *Larixsibirica*, *Malusbaccata*, *Populustremula*, *Prunuspadus*, all species of *Salix* and *Ulmus* and all half-dead trees; in the subcanopy – *Acernegundo*, *Larixsibirica*, all species of *Salix* (except for *Salixcaprea*) and *Viburnumopulus*. Biomass for *Alnusincana* (including the mortmass of downed boles of this species) and *Populustremula* was estimated using literature data (Usoltsev et al. 2018).

The mortmass of the downed bole (P_mort_) was calculated, based on the volume estimations:

**P_mort_=V_mort_ × p_mort_** ,

where P_mort_ – absolute dry mortmass, kg; V_mort_ – the volume of downed bole, m^3^; p_mort_ – average density of moderately decomposed downed bole, kg/m^3^. The data on downed bole densities were taken as constants from literature sources (Klimchenko et al. 2011) for dead pine (constant for downed bole of coniferous trees, 0.307 kg/m^3^) and birch (constant for downed bole of deciduous trees, 0.428 kg/m^3^), as these species predominate in the territory of our study.

**A detailed description of methods for determining the above-ground phytomass of model trees**

The processing of model trees was carried out in August 2010 outside the sampling plots under consideration according to the following method (Usoltsev and Zalesov 2005).

Model trees of each species were selected in such a way as to cover the entire range of variation in their diameters, from minimum values to maximum values. The model trees were felled in August 2010 when the foliage/needles of the current year were fully formed. After felling, the tree’s length was measured. First, the trunk was divided into ten sections. Then, in the middle of each section, measuring from the butt, discs were cut out making it possible to define the diameter of the trunk in the bark and without bark. These measurements were used to calculate the volumes of the tree’s wood and bark. Next, the bark was removed from the discs taken at relative heights of 20, 50 and 80% of the total trunk height, the wood and bark were weighed separately with an accuracy of 0.1 g, their volume was determined and dried to a constant weight in an oven at 110°C. Then the absolute dry mass of the wood and bark were used to calculate a wood/bark proportion and the absolute dry phytomass of the wood and bark of the entire tree trunk.

The phytomass of tree crowns and their structural parts was determined after dividing the crowns into three sections of the same length, since crowns are heterogeneous in the vertical direction regarding the age and thickness of branches, branch coverage and qualitative composition of needles. After weighing each section of the crown (with an accuracy of 50 g), they were divided into leafy/needled and non-leafy/non-needled branches. Then, a sample (about 0.5 kg) was taken from each section’s leafy/needled part to establish the ratio of needles and skeletal parts. For this purpose, we separated needles from the branches and then separately weighed these components’ mass for each sample (with an accuracy of 1 g). The phytomass of needles and woody parts was determined according to the established ratios for each section and the entire crown. To determine the moisture content and absolute dry weight of needles and branches, samples were taken from each part of the crown and then immediately weighed with an accuracy of 0.01 g. Samples of branches were taken separately from leafy/needled and non-leafy/non-needled branches. The obtained values were used to calculate the absolute dry weight of the needles and tree branches. Weighed portions of needles and branches were dried to constant weight in thermostats at a temperature of 110ºC.

To determine the phytomass of subcanopy trees, they were subdivided into two groups. Firstly, in height, trees less than 0.5 m were fractionated (divided into a trunk, branches and foliage). After that, they were weighed, dried at 110°C to constant weight and the above-ground absolute dry phytomass was determined. From trees more than 0.5 m in height, leafy/needled shoots were cut off with pruning shears and a sample of 100–500 g was taken and weighed; then leaves/needles were removed from the sample and it was re-weighed. Then the leaves/needles and the rest of the sample were dried separately to constant weight, weighed again and the absolute dry matter content in both fractions was calculated. Their values were used to determine the absolute dry weight of the crown of the entire plant. Together with non-leafy/non-needled shoots, the trunk mass was weighed in total, dried at 110°C to constant weight, summed with the crown’s phytomass and then the above-ground absolute dry phytomass was determined.

### Quality control

Plant species identification was carried out mainly in the field; specimens with controversial species affiliation were photographed or placed in a Herbarium and identified later in a laboratory by specialists from the Institute of Plant and Animal Ecology of the Ural Branch of the Russian Academy of Sciences (IPAE UB RAS).

In the following cases, occurrences were identified only to taxonomic ranks of high levels; first, the forest elements related to coarse woody debris (downed boles, fragments of downed boles and stumps). As a rule, these elements are destroyed and reliable determination of their taxonomic affiliation is difficult even down to the family level. Second, forest elements that were absent at the study sites were added to the dataset in order to record the very fact of their absence. In this case, we found it unnecessary to detail more than to the level of the type.

## Geographic coverage

### Description

The studied areas are located in the southern taiga subzone of the Southern Urals, in the vicinity of Karabash (polluted sites) and 20 km south of Karabash (sites with a background level of pollution). The same set of habitats represents both polluted and non-polluted areas: pine, birch and floodplain forests, reed swamp, sparse birch stand, marshy meadow and dump of household waste.

### Coordinates

55.3222 and 55.4993 Latitude; 60.1092 and 60.2733 Longitude.

## Taxonomic coverage

### Description

General taxonomic coverage is 1 phylum, 2 classes, 7 orders, 8 families, 19 genera and 23 species of woody vegetation (Table 4).

It should be noted that the mature birch trees, presented in the dataset, are identified to genus. The birch is the only species which was not identified by biological species, but by genus (Novikova 2016) since different growing conditions determine a large variability of morphological characters in individual species. Vetchinnikova (Vetchinnikova 2004) noted that, “... the phylogeny and relationships of species in the genus Betula are rather complex; therefore its systematics is extremely difficult and requires new methodological approaches”. Nevertheless, young plants of the genus Betula were quickly identified to species by the presence/absence of pubescence on the leaf blades.

It is also worth adding that only a few members of the genus Salix have been identified as species. The overlap of the ranges of many willow species and their close phylogenetic relationship contributed to the emergence of a significant number of natural hybrids in willows (about 90 hybrid forms have been described) (Antsiferov 1984), which significantly complicates the identification of their species.

### Taxa included

**Table:** 

Rank	Scientific Name	
class	Magnoliopsida	
order	Dipsacales	
family	Adoxaceae	
order	Fabales	
family	Fabaceae	
order	Fagales	
family	Betulaceae	
order	Malpighiales	
family	Salicaceae	
order	Rosales	
family	Rosaceae	
family	Ulmaceae	
order	Sapindales	
family	Sapindaceae	
class	Pinopsida	
order	Pinales	
family	Pinaceae	

## Temporal coverage

**Data range:** 2012-7-10 – 2012-7-20.

## Usage licence

### Usage licence

Creative Commons Public Domain Waiver (CC-Zero)

## Data resources

### Data package title

Biomass and mortmass of woody vegetation in metal-contaminated areas (Southern Urals, Russia)

### Resource link

https://ipt.ipae.uran.ru/resource?r=frm_bergman_2012&v=1.4

### Number of data sets

1

### Data set 1.

#### Data set name

Woody vegetation under industrial pollution (Southern Urals, Russia): modifying influence of habitat conditions

#### Data format

sampling event

#### Number of columns

49

#### Download URL

https://www.gbif.org/dataset/61384edd-2d0a-437b-8cf0-ff4d2dfcc0da

#### Data format version

1.4

#### Description

The dataset (Bergman and Nesterkov 2021) includes the results of an assessment of the woody vegetation biomass at seven habitats (pine, birch and floodplain forests, reed swamp, sparse birch stand, marshy meadow and dump of household waste) of areas with different levels of industrial pollution in vicinities of the Karabash, the Southern Urals. Karabash Copper Smelter (KCS) is one of Russia’s most significant point polluters; the main components of its emissions are heavy metals, dust and sulphur dioxide. Parameters of woody vegetation (diameter at breast height, diameter at root collar level and biomass) were estimated for seven forest elements: forest stand, subcanopy, half-dead tree of a forest stand and four types of coarse woody debris (downed bole, fragment of downed bole, standing dead tree and stump) at 41 sampling plots (20 at non-polluted and 21 at polluted areas) and 165 subplots (81 and 84, respectively). The dataset includes 411 sampling events (estimation events of the forest elements at sampling plots and subplots), corresponding to 5786 occurrences (estimations of the woody vegetation components) observed during July 2012. For most woody vegetation components (72%), an estimate of the above-ground phytomass is given. For each sampling event, information on the presence or absence of woody vegetation species at the considered habitats is provided. The dataset can be used for environmental monitoring, sustainable forest management, modelling forest productivity considering global changes, studying the structure and biodiversity of forest cover and assessing forests’ carbon-sequestration capacity. In addition, the dataset provides information about different forest ecosystems under the influence of strong industrial pollution.

**Data set 1.** 

Column label	Column description
eventID	An identifier for the set of information associated with an Event (an identifier of the sampling plot with information about the forest element type encoded).
occurrenceID	An identifier for the Occurrence (an estimation of the forest element parameters with information about the forest element type encoded).
country	The name of the country or major administrative unit in which the Location occurs (Russian Federation).
stateProvince	The specific description of the place (Chelyabinskaya Oblast').
municipality	The full, unabbreviated name of the next smaller administrative region than county in which the Location occurs (Karabash or Miass).
locality	The specific description of the place (Karabash, Novoandreevka or Tyelga).
countryCode	The standard code for the country in which the Location occurs (RU).
ownerInstitutionCode	The name (or acronym) in use by the institution having ownership of the object(s) or information referred to in the record (Institute of Plant and Animal Ecology (IPAE)).
locationID	An identifier for the set of location information.
basisOfRecord	The specific nature of the data record (HumanObservation).
samplingProtocol	The description of the method or protocol used during an Event (a complete estimation of woody vegetation trunk diameters at breast height using a caliper).
samplingEffort	The amount of effort expended during an Event (a time period to estimate parameters of all woody vegetation elements at the sampling plot).
sampleSizeValue	A numeric value for a measurement of the size of a sample in a sampling event (an area of the sampling plot).
sampleSizeUnit	The unit of measurement of the size of a sample in a sampling event (square metres).
occurrenceRemarks	Comments or notes about the Occurrence (a notes on the calculation and availibility of biomass data).
eventDate	The date-time during which an Event occurred.
year	The four-digit year in which the Event occurred, according to the Common Era Calendar (2012).
month	The ordinal month in which the Event occurred (7).
decimalLatitude	The geographic latitude (in decimal degrees, using the spatial reference system given in geodeticDatum) of the geographic centre of a Location.
decimalLongitude	The geographic longitude (in decimal degrees, using the spatial reference system given in geodeticDatum) of the geographic centre of a Location.
geodeticDatum	The ellipsoid, geodetic datum or spatial reference system (SRS) upon which the geographic coordinates given in decimalLatitude and decimalLongitude are based (WGS84).
coordinateUncertaintyInMetres	The horizontal distance (in metres) from the given decimalLatitude and decimalLongitude describing the smallest circle containing the whole of the Location (10 metres).
maximumElevationInMetres	The upper limit of the range of elevation (altitude, usually above sea level), in metres.
habitat	A category of the habitat in which the Event occurred (pine forest, birch forest, floodplain forest, reed swamp, sparse birch stand, marshy meadow and dump of household waste).
locationRemarks	Comments or notes about the Location. The investigated areas are subdivided into "polluted" and "non-polluted"; perimeters of the sampling plots or subplots (in metres) are given.
eventRemarks	Comments or notes about the Event. A forest element within the event (forest stand, subcanopy, half-dead tree of a forest stand, downed bole, fragment of downed bole, standing dead tree and stump).
scientificName	The full scientific name, with authorship and date information.
scientificNameAuthorship	The authorship information for the scientificName formatted according to the conventions of the applicable nomenclaturalCode.
recordedBy	A list (concatenated and separated) of names of people, groups or organisations responsible for recording the original Occurrence.
identifiedBy	A list (concatenated and separated) of names of people, groups or organisations who assigned the Taxon to the subject.
kingdom	The full scientific name of the kingdom in which the taxon is classified.
phylum	The full scientific name of the phylum or division in which the taxon is classified.
class	The full scientific name of the class in which the taxon is classified.
order	The full scientific name of the order in which the taxon is classified.
family	The full scientific name of the family in which the taxon is classified.
genus	The full scientific name of the genus in which the taxon is classified.
specificEpithet	The name of the first or species epithet of the scientificName.
taxonRank	The taxonomic rank of the most specific name in the scientificName.
occurrenceStatus	A statement about the presence or absence of a Taxon at a Location.
measurementValue	The value of the measurement, fact, characteristic or assertion (measurements of the tree trunk diameters).
measurementType	The nature of the measurement, fact, characteristic or assertion (two types of estimations of the tree trunk diameters, the DBH (diameter at breast heght) and the diameter at root collar level).
measurementAccuracy	The description of the potential error associated with the measurementValue.
measurementUnit	The units associated with the measurementValue (centimetre).
measurementDeterminedBy	A list (concatenated and separated) of names of people who determined the value of the MeasurementOrFact (Igor E. Bergman).
measurementMethod	A description of the method or protocol used to determine the measurement, fact, characteristic or assertion ("caliper," "pole caliper" or "caliper | measuring tape").
measurementRemarks	Comments or notes accompanying the MeasurementOrFact (if possible, each forest element's specimen is assigned to decidious or coniferous).
identificationRemarks	Comments or notes about the Identification.
organismQuantity	A number or enumeration value for the quantity of organisms (the biomass of forest element's specimens).
organismQuantityType	The type of quantification system used for the quantity of organisms (kilogram).

## Figures and Tables

**Table 1.** Habitat types and location of the sampling plots (coordinates of centres are given; dash denotes absence of the sampling plot: only two plots were established at the reed swamp of non-polluted area).

**Habitat**	**Non-polluted sites**				**Polluted sites**			
	**Sampling plot**	**Decimal longitude**	**Decimal latitude**	**Elevation, m a.s.l.**	**Sampling plot**	**Decimal longitude**	**Decimal latitude**	**Elevation, m a.s.l.**
Pine forest (PF)	b-PF-1	55.3437	60.2078	296	i-PF-1	55.4282	60.2547	298
	b-PF-2	55.3441	60.2091	320	i-PF-2	55.4280	60.2559	312
	b-PF-3	55.3437	60.2097	307	i-PF-3	55.4272	60.2564	303
Birch forest (BF)	b-BF-1	55.3226	60.2263	366	i-BF-1	55.4993	60.2609	350
	b-BF-2	55.3223	60.2262	335	i-BF-2	55.4991	60.2607	360
	b-BF-3	55.3222	60.2259	361	i-BF-3	55.4985	60.2607	367
Floodplain forest (BlF)	b-BlF-1	55.3436	60.2257	287	i-BlF-1	55.4232	60.2714	279
	b-BlF-2	55.3440	60.2270	294	i-BlF-2	55.4238	60.2717	283
	b-BlF-3	55.3437	60.2281	305	i-BlF-3	55.4232	60.2733	276
Reed swamp (BB)	b-BB-1	55.3365	60.1823	297	i-BB-1	55.4565	60.1695	329
	b-BB-2	55.3371	60.1825	296	i-BB-2	55.4564	60.1695	330
	-	-	-	-	i-BB-3	55.4566	60.1697	331
Sparse birch stand (BtF)	b-BtF-1	55.3435	60.2062	296	i-BtF-1	55.4623	60.1432	320
	b-BtF-2	55.3428	60.2055	288	i-BtF-2	55.4621	60.1440	321
	b-BtF-3	55.3427	60.2048	296	i-BtF-3	55.4613	60.1441	324
Marshy meadow (MM)	b-MM-1	55.3426	60.2076	297	i-MM-1	55.4610	60.1108	339
	b-MM-2	55.3424	60.2056	301	i-MM-2	55.4607	60.1101	337
	b-MM-3	55.3433	60.2061	291	i-MM-3	55.4610	60.1092	337
Waste dump (D)	b-D-1	55.3437	60.2171	289	i-D-1	55.4319	60.2498	289
	b-D-2	55.3443	60.2176	290	i-D-2	55.4317	60.2495	292
	b-D-3	55.3435	60.2178	292	i-D-3	55.4322	60.2500	291

**Table 2.** Numbers of sampling plots and subplots in different habitats at non-polluted and polluted areas.

Habitat	Area of pollution	Number of sampling plots	Number of subplots at each sampling plot	Size of subplots, m	Remarks
Birch forest (BF)	Non-polluted	3	9 (3 × 3)	5 × 5	
	Polluted	3	9 (3 × 3)	5 × 5	Heaps of dry branches, data on which are not included in the dataset.
Pine forest (PF)	Non-polluted	3	9 (3 × 3)	5 × 5	
	Polluted	3	9 (3 × 3)	5 × 5	The local population probably withdraws some downed boles.
Floodplain forest (BlF)	Non-polluted	3	9 (3 × 3)	5 × 5	
	Polluted	3	9 (3 × 3)	5 × 5	Some downed boles with saw marks.
Reed swamp (BB)	Non-polluted	2	6 (2 × 3)	5 × 5	
	Polluted	3	9 (3 × 3)	5 × 5	
Sparse birch stand (BtF)	Non-polluted	3	30 (3 × 10)	1 × 1	Periodic grass mowing. Heaps of dry branches not included in the dataset.
	Polluted	3	30 (3 × 10)	1 × 1	
Marshy meadow (MM)	Non-polluted	3	15 (3 × 5)	13 × 6	Periodic grass mowing.
	Polluted	3	15 (3 × 5)	13 × 6	
Waste dump (D)	Non-polluted	3	3 (3 × 1)	8.4 × 4.0	
	Polluted	3	3 (3 × 1)	8.4 × 4.0	

**Table 3.** Constants (a_1_ and a_2_) of regression equations for woody vegetation species of the different forest elements (constants are selected by Levenberg-Macwardt algorithm; R^2^ – coefficient of determination, n – number of sampling units (trees)).

Forest element	Species	а_1_	а_2_	R^2^	n	Collected at	Source
Subcanopy	*Chamaecytisusruthenicus*	0.040593	2.17829	0.94	17	Chelyabinskaya oblast', Southern taiga	Usoltsev et al. 2012
	*Sorbusaucuparia*	0.025749	3.28474	0.99	12	Chelyabinskaya oblast', Southern taiga	Usoltsev et al. 2012
	*Sorbusaucuparia* (large trees)	0.050513	2.69275	0.99	4	Sverdlovskaya oblast', Southern taiga	Usoltsev et al. 2018
	*Rubusidaeus*	0.01467	2.00523	0.71	6	Sverdlovskaya oblast', Southern taiga	Usoltsev et al. 2012
	*Rosa majalis*	0.060674	2.78288	0.95	10	Chelyabinskaya and Sverdlovskaya oblast', Southern taiga	Usoltsev et al. 2012
	*Pinussylvestris*	0.017878	2.92874	0.97	27	Chelyabinskaya oblast', Southern taiga	Usoltsev et al. 2012
	*Betula* sp.	0.023576	3.15147	0.96	18	Chelyabinskaya oblast', Southern taiga	Usoltsev et al. 2012
	*Populustremula*	0.03445	3.08644	0.90	12	Chelyabinskaya oblast', Southern taiga	Usoltsev et al. 2012
	*Alnusincana*	0.017657	3.27133	0.99	7	Chelyabinskaya oblast', Southern taiga	Usoltsev et al. 2012
	*Abiessibirica*	0.052977	2.40134	0.96	23	Sverdlovskaya oblast', Southern taiga	Usoltsev et al. 2012
	*Prunuspadus*	0.065709	1.90958	0.99	7	Chelyabinskaya oblast', Southern taiga	Usoltsev et al. 2012
	*Cotoneasterlucidus*	0.041403	2.73328	0.78	8	Chelyabinskaya oblast', Southern taiga	Usoltsev et al. 2012
	*Salixcaprea*	0.033553	2.67063	0.99	3	Sverdlovskaya oblast', Southern taiga	Usoltsev et al. 2012
Forest stand	*Betula* sp.	0.098613	2.53413	0.97	56	Chelyabinskaya oblast', Southern taiga	Usoltsev et al. 2012
	*Pinussylvestris*	0.12004	2.38449	0.97	40	Chelyabinskaya oblast', Southern taiga	Usoltsev et al. 2012
	*Piceaobovata*	0.321051	2.04809	0.97	33	Chelyabinskaya oblast', Southern taiga	Usoltsev et al. 2012
	*Populustremula*	0.14492	2.30065	0.99	5	Southern Karelia, Middle taiga	Usoltsev et al. 2018
	*Alnusincana*	0.065389	2.4907	0.99	17	Vologodskaya oblast', Middle taiga	Usoltsev et al. 2018
Standing dead trees	*Betula* sp.	0.12954	2.39547	0.99	56	Chelyabinskaya oblast', Southern taiga	Usoltsev et al. 2012
	*Pinussylvestris*	0.130432	2.31176	0.96	40	Chelyabinskaya oblast', Southern taiga	Usoltsev et al. 2012
	*Alnusincana*	0.068865	2.47813	0.99	5	Southern Karelia, Middle taiga	Usoltsev et al. 2018

**Table 4.** Distribution of occurrences with the lowest taxon rank identified

**Group of forest elements**	**Forest element**	**Taxon rank**					
		**Phylum**	**Class**	**Family**	**Genus**	**Species**	**Total**
Forest stand	Forest stand				498	745	**1243**
	Half-dead tree of a forest stand	33			19	11	**63**
Subcanopy	Subcanopy				14	3410	**3424**
Coarse woody debris	Standing dead tree	19			217	91	**327**
	Downed bole	25	144	9			**178**
	Fragment of downed bole	30	52	3			**85**
	Stump	451	15				**466**
**Total**		**558**	**211**	**12**	**748**	**4257**	**5786**
